# An Optical Fibre-Based Sensor for Respiratory Monitoring

**DOI:** 10.3390/s140713088

**Published:** 2014-07-21

**Authors:** Marek Krehel, Michel Schmid, René M. Rossi, Luciano F. Boesel, Gian-Luca Bona, Lukas J. Scherer

**Affiliations:** 1 Empa, Swiss Federal Laboratories for Materials Science and Technology, Laboratory for Protection and Physiology, Lerchenfeldstrasse 5, 9014 St. Gallen, Switzerland; E-Mails: marek.krehel@empa.ch (M.K.); michel.schmid@empa.ch (M.S.); rene.rossi@empa.ch (R.M.R.); gian-luca.bona@empa.ch (G.-L.B.); lukas.scherer@empa.ch (L.J.S.); 2 ETH Zurich, Swiss Federal Institute of Technology, Department of Information Technology and Electrical Engineering, Gloriastrasse 35, 8092 Zurich, Switzerland

**Keywords:** breathing monitoring, fibre optic sensing, respiratory rate, medical textiles, photonic textiles, technical textiles

## Abstract

In this paper, a textile-based respiratory sensing system is presented. Highly flexible polymeric optical fibres (POFs) that react to applied pressure were integrated into a carrier fabric to form a wearable sensing system. After the evaluation of different optical fibres, different setups were compared. To demonstrate the feasibility of such a wearable sensor, the setup featuring the best performance was placed on the human torso, and thus it was possible to measure the respiratory rate. Furthermore, we show that such a wearable system enables to keep track of the way of breathing (diaphragmatic, upper costal and mixed) when the sensor is placed at different positions of the torso. A comparison of the results with the output of some commercial respiratory measurements devices confirmed the utility of such a monitoring device.

## 1. Introduction

The respiratory rate is the frequency of breaths taken within a certain amount of time. This rate can vary depending on the need for oxygen. For instance, if the resting body starts to work, the respiratory rate increases. The respiratory rate is correlated to the gas composition in the blood, e.g., faster breathing can occur when the oxygen level is low and the carbon dioxide or the carbon oxide level is high. This means that an increased breathing rate can be caused by an increased carbon oxide concentration deriving from an infection [1]. Abnormal breathing rates can also be caused by other specific medical conditions that require medical attention, e.g., fever, dehydration, lung cancer, use of narcotics, alcohol abuse, *etc.* [2]. Three types of breathing can be differentiated. (1) Diaphragmatic breathing: the main expansion is in the abdominal and lateral costal parts; (2) Upper costal breathing: chest expands the most; and (3) a mix of diaphragmatic and upper costal breathing. The first one is considered as optimal breathing since lungs can expand the most and thus the highest gas exchange can take place [3,4]. Since the three ways of breathing lead to the expansion of different parts of the human torso, they can be distinguished by measuring the pressure change at different places of the torso.

To be able to better analyse and interpret the breathing rate, a solution for long-term monitoring is required. Attempts of monitoring systems lacked the desired comfort for patients and were thus not suited for long-term use [5].

A fibre optic fabric-based sensor system is ideally suited for long-term monitoring due to its enhanced comfort and versatility [6]. The other advantages of optical fibres-based sensors compared to electrical or chemical devices are their insensitivity to electromagnetic fields, water and corrosion resistance, and their compact size combined with low small weight [7,8]. The insensitivity to electromagnetic fields is of great importance in the hospital environment where a lot of such fields are present [9]. This opens the door to the use of fibre optic fabrics during magnetic resonance imaging (MRI) and computed tomography (CT) examinations [7,10–13]. Recently researchers successfully developed MRI compatible sensors, for instance by means of a Plexiglas springboard which converts patients' movements to strain, and the latter is then measured by a fibre Bragg grating system [14–16]. Different approaches to measure the respiratory rate with optical fibres have been studied. Zieba *et al.* recorded changes in the distance between the light waveguide end and a sensor's head, which were caused by the chest movements [17]. Within the Seventh Framework Programme “Ofseth”, optical fibres-based optical time-domain reflectometry for distributed respiration measurement and optical fibres with long period gratings in microstructured POF were evaluated. They were incorporated into elastic fabrics to measure the breathing rate and the breathing volume [18–20]. Similar principles have been reported and a summary presented in [19]. Augousti *et al.* proposed a respiratory analysis by means of macro bending loss effects [21,22]. Although most of the published work dealing with textile-based breathing monitoring systems show satisfactory sensing capabilities, their use in a medical environment is still limited by the poor usability of the sensors for the medical staff and the patient [19]. Furthermore, the high costs and the incompatibility with current industrial textile processes have hindered market entrance. The brittleness and the weak mechanical strength of optical glass fibres is an additional challenge when it comes to the textile integration of these fibres using an industrial process. Yoo *et al.* also showed respiratory rate measurements systems, but their systems, even if capable of being integrated into textiles, would be of very low haptic response. They used commercial optical fibers with additional springs and mirrors [23]. For wearable systems, simple and miniaturized electronics with small power consumption are necessary.

So far no one has reported a textile-based solution that would measure the respiratory rate by means of signal intensity changes and that would enable to distinguish between the three types of breathing. The elastomeric waveguides, which we have reported elsewhere [24], overcome the abovementioned comfort and industrialization problems. These waveguides react to applied pressure with a change in light intensity. In this study, we demonstrate the feasibility of using these elastic and flexible waveguides to measure the respiratory rate and to evaluate the type of breathing.

## 2. Experimental Section

### 2.1. Materials and Methods

**Figure 1.**
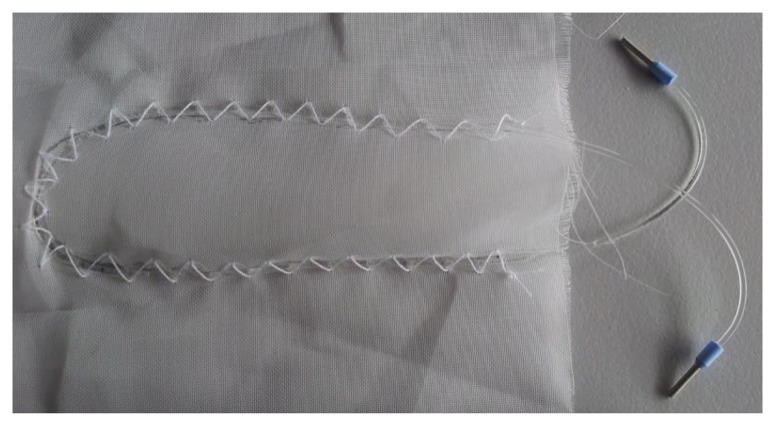
Picture illustrating the integration of the fibre into the textile to form the sensing setup, herein called “half oval”.

Four custom made optical fibres, varying in flexibility, were used to perform the measurements (Geniomer (Gm) 100 HDS d = 0.5 mm, Gm 100 d = 0.75 mm, Gm 100 HDS d = 0.8 mm and Gm 175 d = 0.5 mm). The optical fibres had attenuation parameters between 0.16 dB/cm–0.25 dB/cm at 652 nm. Moreover, their high yield strength (3.9–5.4 MPa) and flexibility allowed to manually integrate them into textiles. Comprehensive information about production and the characteristics of these fibres can be found in [24]. In order to integrate the fibres into a textile as illustrated in Figure 1, they were laced in a woven polyester textile, provided by Engelbert E. Stieger (Rorschacherberg, Switzerland).

**Figure 2.**
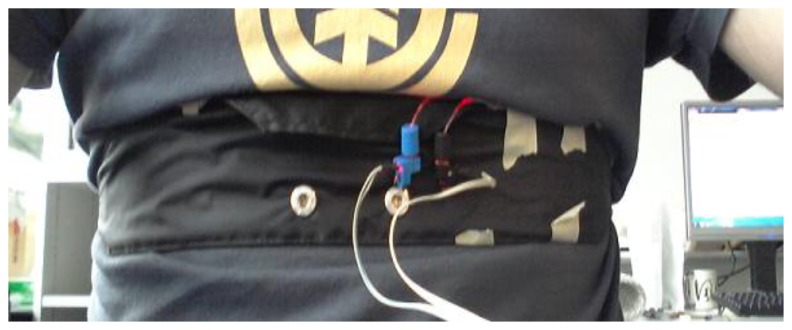
Sensing system placed on a human subject study. Blue and black connectors are housings for the LED and the photodiode, respectively. The power supply is provided by the grey wires.

The sensing textiles were sewed on the inside of a custom developed chest strap which consisted of polyester textile, rubber straps and a buckle. Afterwards the strap was placed on a test subject (28 year old, male, 182 cm, 72 kg) as shown in Figure 2.

**Figure 3.**
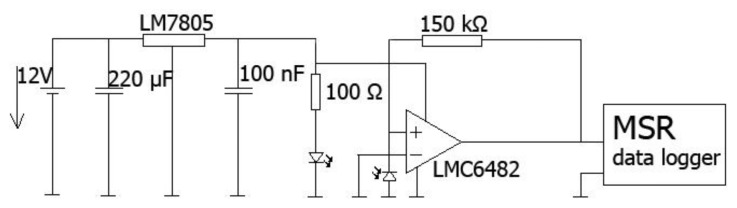
Sketch illustrating the electronics used to power the LED, and to amplify and to log the signal from the photodiode.

The scheme from Figure 3 shows the driving and measuring electronics. As a light source, a red LED (660 nm) housed in connector (IFE99B) provided by I-Fiberoptics (Tempe, AZ, USA), and a photodiode, enclosed in a connector (IFD91) with housing dimensions of 24 mm × 7 mm, provided by the same company to detect the remaining light reaching the other end of the optical fibre. The device package features a micro lens for efficient light coupling. A 150 kΩ resistor was used to provide the gain in the signal and was changed depending on the signal strength. The MSR Data logger had a sampling frequency of 50 Hz. Total weight of light sources and electronics was below 100 g.

All the acquired data were evaluated in MATLAB R2012b.

#### 2.1.1. Comparison of the Fibre Types

The performance of all four types of fibres was conducted in two scenarios, first with a slow breathing frequency (∼6 breaths per minute), and secondly with a fast breathing frequency (∼42 breaths per minute). The logged data was band pass filtered for respiratory rates between 5 and 60 respiratory cycles per minute. The measurements were all conducted with half oval form geometry of the fibre (Figure 1).

#### 2.1.2. Comparison of Different Fibre Setups

**Figure 4.**
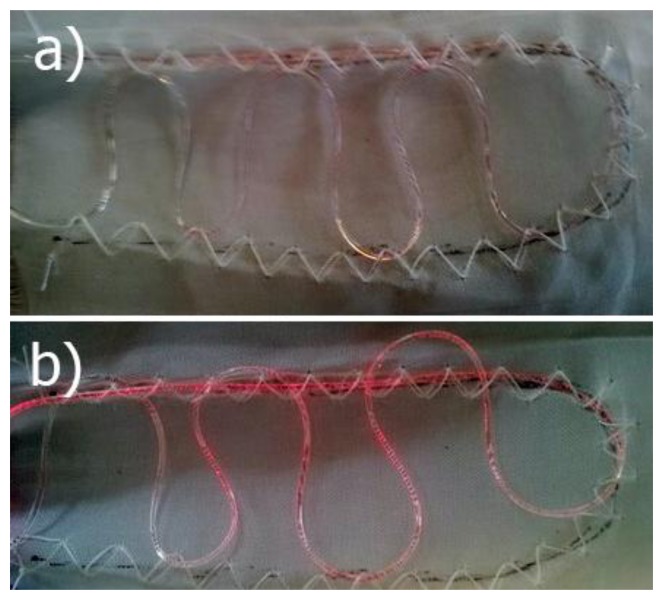
Picture presenting modification of half oval form. In (**a**) fibre was folded without cross points; whereas in (**b**) fibre crossing took place.

Different setups from fibre Gm 175 d = 0.5 mm were tested. Firstly, the half oval form, as presented in Figure 1, with three different fibre lengths (240 mm, 180 mm, and 60 mm) were prepared. Afterwards, the half oval shape was additionally arranged with an additional meander consisting of 6 bends in the middle of the half oval, as presented in Figure 4. Two variations of this setup were prepared. In the first setup (shown in Figure 4a) the fibre was integrated without cross points; in the second setup (Figure 4b) the sensing fibre had six cross points (at the bends of each meander with the half oval).

The last setup was a straight fibre wrapped around the chest with a chest strap (described in Section 2.1). The measuring setup was the same as described in that section. Due to the weaker signals in these tests, the gain resistor was increased to Rgain = 1 MΩ. The measurement procedure was the same as described in Section 2.1.1. In Table 1 all the tested fibre setups with their abbreviation are listed.

#### 2.1.3. Comparison of the Sensing Positions

After having determined the type of optical fibre and the setup, the best position of the sensing textile on the human body was assessed. Following breathing scenarios were conducted to compare the sensing positions. Firstly, diaphragmatic and upper costal breathing (∼6 breaths per minute) was performed, in which the human subject was asked to inhale a maximum possible amount of air. Secondly, the subject was asked to conduct upper costal breathing (∼15 breaths per minute) under normal resting conditions. Thirdly diaphragmatic breathing (∼12 breaths per minute) was performed.

This procedure was executed at different positions of the sensor on the torso. Three positions were on the front body: on a heart region (1); above the navel (2); above the left hipbone (3); as well as two on the back: below the left shoulder blade (4) and in the center of the back (5).

#### 2.1.4. Cross Comparison with Commercial Device

Measurements from different positions on human torso were additionally verified with a reference measurement. Correlation measurements were conducted by the cardiopulmonary exercise testing instrument (SN808861, CareFusion, San Diego, CA, USA). While conducting cross comparison measurements, the following respiratory rates were monitored: 2 min of normal breathing (∼15 breaths per minute), then 1 min fast breathing (∼50 breaths per minute), followed by 1 min normal breathing. We also analysed the agreement between the two devices with Bland-Altman plots [25]. For that, we took several data points during the 4 min experiment. As the individual data points were not acquired at the same time intervals, interpolation of some of the data was required to create the plot.

## 3. Results and Discussion

### 3.1. Comparison of the Sensitivity of Different Optical Fibres

In order to determine the best suited fibre, all the samples were evaluated under slow and fast breathing conditions. Additionally, this allowed checking if the sampling frequency of the sensing system was fast enough. Normal respiratory rates for a healthy adult varies between 10 and 20 respirations per minute [26]. However, it can easily rise while e.g., performing sport activities [27]. Therefore, the sensing system was tested in the respiration rate that goes well beyond this number (respiratory rate frequencies of around 6 and of 50 breaths per minute were evaluated as well).

**Figure 5.**
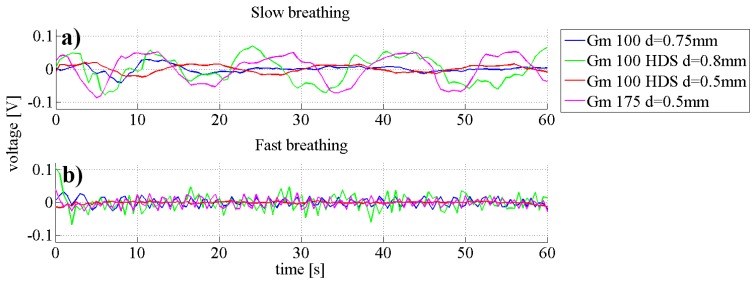
Graphs representing the slow breathing (**a**) and fast breathing (**b**) scenario. All four fibres types were measured for the two scenarios.

The quality of measurements is determined by the signal to noise ratio, which in turn means the stronger the signal the better the quality of the data output will be. In Figure 5 the measurements of two breathing conditions with all four types of fibres are presented. It was observed that changes in signal intensity in waveguides Gm 100 d = 0.75 mm and Gm 100 HDS d = 0.5 mm were very low for the slow breathing scenario (0.024 ± 0.06 V for Gm 100 d = 0.75 mm and 0.015 ± 0.03 V for Gm 100 HDS d = 0.5 mm). Both fibres Gm 100 HDS d = 0.8 mm and Gm 175 d = 0.5 mm exhibited higher signals (with amplitudes in the range of 0.06 ± 0.01 V for Gm 100 HDS d = 0.8 mm and 0.048 ± 0.03 V under slow breathing). In the fast breathing scenario, the signal from fibre Gm 100 HDS d = 0.5 mm had no detectable peaks. Signal changes from fibres Gm 100 d = 0.75 mm and Gm 175 d = 0.5 mm were comparable and varied between 0.02 and 0.03 V. Highest signal amplitudes (while fast breathing) were obtained from fibre Gm 100 HDS d = 0.8 mm. However, it was noticed that the measurement signals from fibre Gm 100 HDS d = 0.8 mm featured more interfering frequencies under slow and fast breathing. Therefore, fibre Gm 175 d = 0.5 mm was chosen for further experiments due to the high signal strength and the lowest interfering frequencies.

### 3.2. Comparison of Different Sensor Setups

The same tests described in Subsection 3.1. were conducted to determine the best geometry of the textile-integrated optical fibre. In order to only use one electronic device with the LED and the photodiode close to each other, the two fibre ends were always close to each other. Firstly, the samples with the half oval geometry were examined (fibre lengths of 240 mm, 180 mm and 60 mm, respectively). Different lengths were measured in order to find out what is the best length of the sensing waveguide that could be used. Secondly, the setup with additional bends was studied (HOAB).

It was expected that the additional bends would have a beneficial input on the sensitivity, because more fibre surface was in touch with the skin surface. Afterwards the setup HOCF was studied. The setup HOCF differed from HOAB in that each of the additional bends from the meander crossed the main loop twice, which means the fibre had in total six cross points. Due to the additional pressure applied on these cross points, the sensor was expected to be more sensitive. Lastly, the setup with the waveguide wrapped around the chest was studied (FAC). This was the longest sensing waveguide in contact to the skin and since it surrounded the torso, breathing volume should be determinable with this sensing geometry.

**Figure 6.**
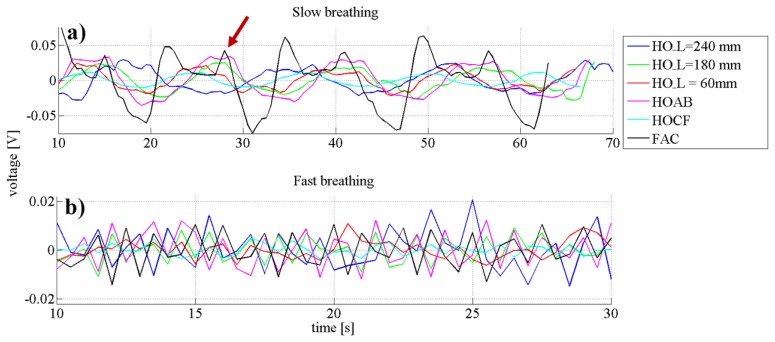
Graphs representing the data required to evaluate which sensor form is the best suited for respiratory rate measurements. On top slow breathing and on the bottom fast breathing.

In Figure 6a, the plots from different sensor setups when the subject was breathing slowly are presented. The signal strength for the FAC setup was the highest due to the amplification with a stronger resistor since the signal with the resistor used for the other fibre geometries was at the detection limit (1 MΩ *vs.* 1 kΩ, which makes a 6.7 times higher gain). This amplification also increased the artefacts, which were present as additional peaks that did not correspond to the respiratory rate. One prominent artefact (indicated by red arrow) signal appeared always a 5 s after the main breathing signal. It was a result of a back pressure applied against the back of the chair, and it was not present in the other scenarios since the sensing fibres were placed in front of the torso and thus no pressure was applied when the body was pressed against the chair. The signal from the sample HOCF had the lowest amplitude (0.015 V). This can be explained by the additional bends and crossing fibres, which highly attenuated the signal, whereas all other sensor setups showed very similar amplitude changes.

In Figure 6b the graphs of the fast breathing scenario are presented. The plotting from the HOL_ = 60 mm was nearly flat and no peaks that would correspond to the respiratory rate were detected. All the other samples had similar amplitude changes in the range of 0.010 ± 0.03 V.

Since the sensing textile HO_L = 180 mm had the lowest amount of interfering signals in slow breathing and had a comparable amplitude difference as the other samples while fast breathing, this geometry was chosen for further examinations.

### 3.3. Positioning of Sensors to Distinguish Different Types of Breathing

**Figure 7.**
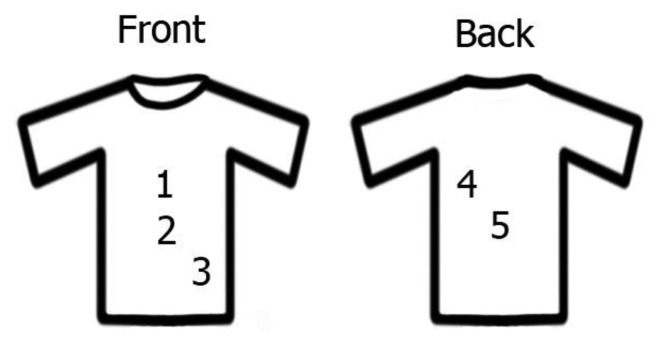
Positions of setup placed on subject study. On the front side: (1) on a heart region; (2) above the navel; (3) above the left hipbone; and on the back side: (4) below left shoulder blade; (5) in the middle of the back.

In order to find out what is the best place on human body to measure respiratory rate the measurements of three types of breathing types (diaphragmatic, upper costal and mixed breathing) were evaluated at five different positions on the human body (Figure 7).

**Figure 8.**
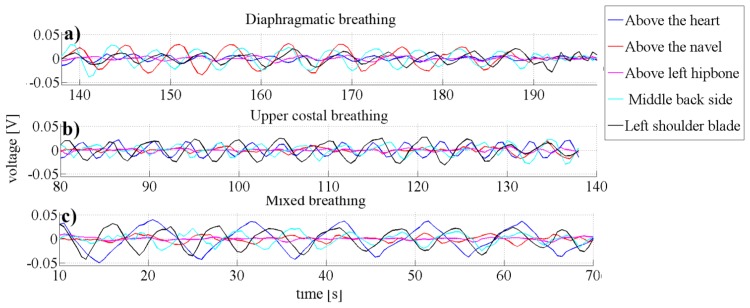
Graphs representing data from different types of breathing and data required to find out what is the best place on human body to measure respiratory rate.

Figure 8 compares the measurements from the above-mentioned breathing types. Figure 8a presents the measurements while the subject was performing diaphragmatic breathing, the best signal change was obtained when the sensor setup was placed above the navel (position 2) (0.027 ± 0.003 V). Signal artefacts were not observed. As discussed in the introduction, the girth around the abdominal expands most during this type of breathing. This explains the highest detected pressure change while breathing when the sensor was placed on position 2. Peaks related to breathing frequency could also be detected when the sensor was placed on the middle of the back (position 5) with signal amplitudes of around 0.022 ± 0.04 V, interfering frequencies were not noticed in this scenario neither. Signal from setup placed at position 4 could be used for measuring the respiratory rate, however with very small amplitudes (0.013 ± 0.03V). No peaks corresponding to respiratory rate were found when the sensor was placed either above the heart (position 1) or above the hipbone (position 3).

Except setups placed on the position 1 the sensors reacted similar while upper costal breathing was performed (Figure 8b) as they did while diaphragmatic breathing, only with smaller amplitudes. The signal amplitude for sensors placed above the heart (position 1) was 0.017 ± 0.003 V and 0.021 ± 0.004 V for the sensor when placed below the left shoulder blade (position 4). The signal changes when the sensor was placed below the center of the torso (positions 2 and 3) were below 0.01 V and no respiratory related peaks could be detected. The low signal was expected, because the body changes the geometry during chest breathing mainly on the upper half of the torso (positions 1, 4 and 5).

It was ascertained that different types of breathing measured at the same place influence the amplitude of the signal. During mixed breathing presented in Figure 8c, the highest signal amplitude (0.036 ± 0.002 V) was obtained from the sensor placed above the heart (position 1). Furthermore, no interfering frequencies were observed. When the sensor was placed at position 4, slightly lower and artefact-free signals (0.025 ± 0.004 V) were measured. Weak signal changes were also observed from the sensor placed in the middle of the back-side (position 5), however the amplitude changes were not constant over time, which hinders long-term breathing monitoring. No peaks belonging to the breathing were observed from sensors placed above the navel (position 2) and above the left hipbone (position 3).

To summarize, the positioning of the sensor has an influence on the signal amplitude and can therefore be used to distinguish the different types of breathing. For instance, when placing one sensing setup above the navel (position 2) and a second one above the heart (position 1) diaphragmatic breathing (position 2) can be distinguished from costal breathing (position 1). When both of these sensors react to respiratory changes, a mixed type of breathing is performed.

### 3.4. Cross Comparison with Commercial Device

The respiratory rate under regular circumstances (no infections, no sport activities) varies between 10 and 20 breaths per minute [26]. However, special events can elevate the breathing frequency close to 60 times per minute [27], and thus the results from our system were compared with a commercial, non-portable system in the range from 10 to 60 breaths per minute.

**Figure 9.**
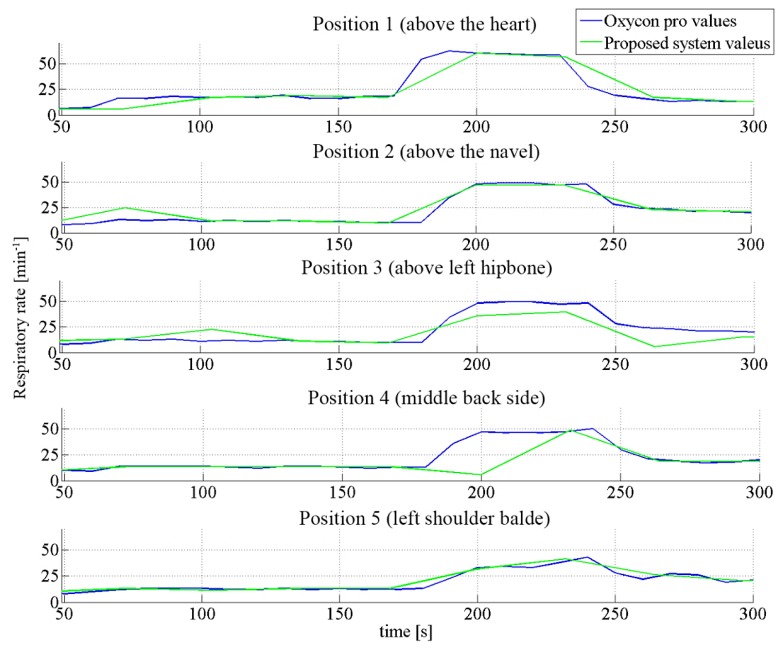
Cross comparison of respiratory data sets from our sensing system and from commercial device.

Figure 9 shows the collected data. The data sets arising from the commercial oxycon device correlated well with the one of the developed sensor. Looking at the “Above the heart” and “Middle back side” graphs, a delay in correlation can be noticed. This slight difference can be explained by the lower time resolution used to calculate the frequency using the short-time Fourier transform (STFT) to extract the respiratory rate from the measured data. A higher frequency resolution was chosen which led to the lower time resolution [28]. For long-term applications, the used time resolution is adequate since lowering of the power consumption is always a subject of wearable systems.

The low signal to noise ratio resulted in a slight underestimation of the frequency in the measurement setup placed above the left hipbone (50 *vs.* 40 breaths per minute) because not all breaths were recorded. It also underlines the results discussed in Section 3.3 where only for diaphragmatic breathing enough signal was obtained when the sensor was placed above the left hipbone.

**Figure 10.**
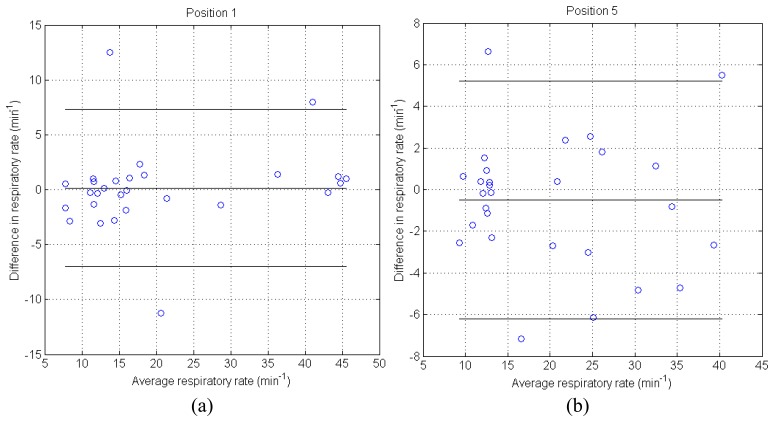
Bland-Altman plots comparing the results from respiratory rate of the oxycon device and our optical fibre sensor. Data taken from Figure 9. The three horizontal lines represent the mean ± 1.96 SD (standard deviation).

Besides the discussed disagreement, the data correlate well in both slow and fast (respiratory rate frequency from 10 to 50 breaths per minute) scenarios. This is shown in Figure 10 for 2 of the 5 studied positions. We observe that the difference in both signals (from the oxycon device and the optical fibre sensor) is roughly constant over the whole duration of the experiment (4 min). Moreover, the differences in respiratory rate for these two positions are mostly concentrated in the range of ±3 min^−1^ (with the exception of a few outliers).

## 4. Conclusions

We demonstrate the feasibility of a fully textile, fibre optics based respiratory rate sensor. Comprehensive studies determined the best suited waveguide and integration geometry to create a breathing rate sensing device. Placing the fibre-based sensor on different areas of the human body showed that depending on the way of breathing, the signal amplitude was different at different positions. This opens new possibilities, such as by integrating two or more sensors in e.g., a T-shirt would allow obtaining information not only about the breathing rate, but also about the way of breathing. The obtained measurement data were additionally verified by comparing them with the output of a commercially available device, which showed good correlation. The flexibility of the sensing textile ensures a good haptic response, which for a wearable long-term monitoring system is crucial. The fact that the electronics do not have to be placed directly on the measurement place allows the adjustment of the rigid electronics on places with a lower disruption for the wearer. Motion artefacts were not significant, thus simple filtering was satisfactory. If the sensor needed to be used under conditions with extraordinary movements, a compact size accelerometer would be used for signal to noise improvement. Furthermore, only one wavelength is necessary for the intensity-based sensor, which simplifies the electronics and allows the miniaturization to an accepted level.

## Figures and Tables

**Table 1.** Description of fibre types with their abbreviation.

**Fibre Setup Type**	**Abbreviation**	**R_gain_ [k Ω]**
Half oval, length 60 mm	HO_L = 60mm	150
Half oval, length 180 mm	HO_L = 180mm	
Half oval, length 240 mm	HO_L = 240mm	
Half oval with additional bends	HOAB	
Half oval with additional bends and crossing fibres	HOCF	

Fibre around the chest	FAC	1000
